# Dystonia Versus Redox Balance: A Preliminary Assessment of Oxidative Stress in Patients

**DOI:** 10.3390/antiox14091052

**Published:** 2025-08-27

**Authors:** Jan Koptielow, Emilia Szyłak, Anna Koptielowa, Magdalena Sarnowska, Katarzyna Kapica-Topczewska, Edyta Adamska-Patruno, Katarzyna Socha, Jan Kochanowicz, Alina Kułakowska, Monika Chorąży

**Affiliations:** 1 Department of Neurology, Medical University of Bialystok, 15-276 Bialystok, Poland; 2 Department of Nutriomics, Clinical Research Centre, Medical University of Bialystok, Marii Sklodowskiej-Curie 24A, 15-276 Bialystok, Poland; 3 Clinical Research Support Centre, Medical University of Bialystok, Marii Sklodowskiej-Curie 24A, 15-276 Bialystok, Poland; 4 Department of Bromatology, Faculty of Pharmacy with the Division of Laboratory Medicine, Medical University of Białystok, Mickiewicza 2D, 15-222 Białystok, Poland; katarzyna.socha@umb.edu.pl

**Keywords:** serum biomarkers, copper, selenium, zinc, TAS, TOS, OSI

## Abstract

Dystonia is defined as a movement disorder, the etiology of which may be linked to oxidative stress. The objective of this study was to evaluate the serum levels of zinc, copper, and selenium, as well as oxidative stress parameters, in patients diagnosed with focal dystonia, in comparison to a control group. The study comprised 39 patients and 30 healthy subjects. Patients demonstrated a marked decrease in Total Antioxidant Status (TAS) (*p* = 0.0002) and an increase in Total Oxidant Status (TOS) and Oxidative Stress Index (OSI) (both *p* < 0.0001), suggesting a redox imbalance. Of the elements examined, only copper exhibited a significant elevation (*p* = 0.0061), while zinc and selenium levels remained unchanged. The results of this study suggest that oxidative stress may play a significant role in the pathophysiology of dystonia and represent a potential target for adjunctive therapy.

## 1. Introduction

Dystonia is a complex neurological disorder that falls into the category of movement disorders. It is characterized by the occurrence of abnormal, repetitive movements and forced postures, which result from involuntary muscle contractions. These conditions can manifest as either continuous or episodic, frequently resulting in a substantial decline in the patient’s quality of life. The occurrence of dystonia is not contingent on age; it has the capacity to affect both children and adults. In many cases, the etiology of the condition is unclear, and it is known as idiopathic or primary dystonia [1].

The classification of dystonias is dependent upon the extent of the symptoms and is categorized as follows: focal, segmental, multifocal, hemidystonia, and generalized dystonia. The most prevalent clinical manifestation is focal dystonia, which involves a specific region of the body, such as the neck, face, hand, or larynx. The segmental form of the condition involves two adjacent regions, and in cases of multifocal dystonia, more than one area that does not belong to a single anatomical location. Hemispheric dystonia is characterized by the unilateral involvement of one body side, while generalized dystonia typically originates in the lower limbs and subsequently extends to other body regions [2].

The most prevalent forms of focal dystonia are as follows:

- Cervical dystonia (CD) is the most prevalent form of focal dystonia. It is characterized by involuntary contractions of the neck and shoulder muscles, which result in compulsive head movements, tremors, and pain. Cervical dystonia is a condition that is characterized by the presence of pain, which can be both common and debilitating. This pain can have a significant impact on the patient’s social and occupational functioning [3,4].
- Blepharospasm (BSP) is characterized by involuntary closure of the eyelids due to contractions of the eye’s circular muscle. This condition can result in partial or complete functional blindness, despite the presence of normal visual acuity [5,6].
- Oromandibular dystonia (OMD) is a condition that affects the muscles of the face, tongue, and mandible. The condition is characterized by involuntary mouth opening and closing, protrusion of the tongue or jawbone, and potentially leads to oral trauma and difficulties with speech and mastication [7].
- Spasmodic dysphonia (SD) is a form of laryngeal dystonia, defined as a condition in which spasmodic contractions of the laryngeal muscles interfere with the production of speech. A distinction is made between adduction type (the most common type, producing a ‘squeezed’ voice) and abduction type (producing a weak, husky voice) [8].
- Focal hand dystonia (FHD) is characterized by the manifestation of task-dependent dystonia, a condition that can be exemplified by various forms, including writer’s dystonia and musician’s dystonia. This manifests as difficulty controlling precise hand movements, which can prevent people from pursuing occupations that require high manual precision [9].

The diagnosis of dystonia is primarily based on a comprehensive analysis of the clinical picture, encompassing the patient’s medical history, observation of their posture, and movements. The presence of features such as ‘mirror dystonia’ (the manifestation of symptoms on the opposite side when moving), ‘sensory tricks’ (gestures or positions that bring temporary relief), and the dystonic nature of the tremor is of crucial significance [10].

Despite extensive research, the pathophysiology of dystonia remains incompletely elucidated. Previous theoretical frameworks predominantly concentrated on the dysfunction of the basal nuclei of the brain. However, contemporary theories now place greater emphasis on the role of a sophisticated network of structures, encompassing the cerebral cortex, the cerebellum, and the thalamus. Abnormalities in sensory processing, motor integration, and the functioning of inhibitory systems within the nervous system are central to the development of dystonic symptoms [11,12].

In dystonia, a genetic mutation in the TOR1A gene results in the production of a mutant TorsinAΔE protein. This, in turn, disrupts protein homeostasis within the endoplasmic reticulum and nuclear envelopes, leading to chronic activation of the UPR response, which is characterized by the abnormally folded protein. It has been demonstrated that persistent activation of this pathway can disrupt metabolic processes and the redox balance, with the resultant generation of excess reactive oxygen species [13].

Recent investigations have shown that alterations in serum concentrations of essential trace elements—particularly zinc, selenium, iron, copper, and manganese—are closely linked to dysregulated oxidative stress in neurological disorders [14]. Furthermore, oxidative stress has been identified as a potential mechanism of secondary neuronal damage [15] and may play an important role in the pathogenesis of dystonia. Therefore, we have undertaken an assessment of oxidative stress in patients’ serum, as assessment of oxidative stress in patients’ serum seems reasonable as a potential indicator of molecular abnormalities accompanying dystonia.

## 2. Materials and Methods

### 2.1. Patients

The data collection for this study was conducted with the approval of the Bioethics Committee at the Medical University of Bialystok (approval no. APK.002.6.2023). The research was conducted in accordance with the ethical standards set out in the Declaration of Helsinki and followed the established principles of good medical practice.

A total of 69 participants (control group: 30; dystonia patients: 39) were recruited between 23 January and 15 March 2025. Inclusion criteria were as follows: age between 18 and 75 years, being under the care of the Department of Neurology of the University Clinical Hospital in Bialystok, and provision of written informed consent. Exclusion criteria included hypersensitivity to botulinum toxin type A or any component of the formulation, an active infection or inflammation at the intended injection site, or if they were diagnosed with neuromuscular disorders such as myasthenia gravis, Lambert–Eaton syndrome, or amyotrophic lateral sclerosis (ALS). Additional exclusion criteria included pregnancy or breastfeeding, congenital or acquired coagulation disorders (e.g., hemophilia), and the use of medications that impair neuromuscular transmission, such as aminoglycoside antibiotics (e.g., gentamicin and tobramycin).

All participants provided written informed consent, thereby authorizing the use of their blood test results, dietary survey responses, and clinical examination outcomes for the purpose of scientific publication.

### 2.2. Sample Preparation

Dietary information was collected using a food frequency questionnaire developed by the Committee of Human Nutrition Science of the Polish Academy of Sciences. Patients diagnosed with dystonia were invited to complete a questionnaire designed to assess their consumption frequency of a range of food products. The process of completing the questionnaire was overseen by a physician who was responsible for the supervision of care in the neurology department. The questionnaire included 34 distinct food categories, covering a wide range of items such as various types of bread, cereals, dairy products, meats, fish, fats, vegetables, fruits, sweets, beverages, and alcoholic drinks. For the purposes of this study, food consumption was classified as frequent if the product was consumed at least two to three times per week, or at least once a week in the case of fish. The consumption of each item was categorized as infrequent if it was consumed no more than once per week. These classifications are in accordance with the standards recommended by the Committee on Human Nutrition Science of the Polish Academy of Sciences.

Blood samples, with a volume of approximately 8 mL each, were obtained from participants using vacutainer tubes with clot activator designed for elemental analysis (Becton Dickinson, Rungis, France). Following collection, the samples were subjected to a centrifugal process at approximately 1000× *g* for a duration of 10 min, with the objective of effectuating the separation of the serum. Thereafter, the serum samples were stored under refrigerated conditions at a temperature of −20 °C. In order to prepare the serum for elemental analysis, deproteination was performed using 1 mol/L spectral-grade nitric acid (Merck, Darmstadt, Germany). Subsequently, a 1% solution of Triton X-100 was added to the samples, which were then vortexed and subjected to a second centrifugation process for a duration of 10 min. The resulting supernatant was then analyzed for zinc concentrations. The assessment of copper content was conducted following appropriate dilution in 0.1 M nitric acid, while selenium concentrations were determined after a 1:1 dilution with 0.2% Triton X-100.

### 2.3. Measurement of Mineral Components and Total Antioxidant Status

The concentrations of selenium (Se), copper (Cu), and zinc (Zn) in serum samples were measured using atomic absorption spectrometry. Electrothermal atomization was employed for the determination of Se and Cu levels, while a flame atomization method was used for Zn. All analyses were performed using the Z-2000 spectrometer (Hitachi, Tokyo, Japan) with Zeeman background correction. The specific wavelengths employed for the detection of Se, Cu, and Zn were 196 nm, 324.8 nm, and 213.9 nm, respectively. The calibration process was executed by utilizing working standards that were prepared from 1 g/L stock solutions supplied by Merck (Darmstadt, Germany). The detection limits were established at 1.84 μg/L for Se, 0.51 μg/L for Cu, and 0.011 mg/L for Zn. In order to ensure the reliability and validity of the measurements obtained, a certified reference serum material (Seronorm Trace Elements, Serum Level 1, 0903106; Sero AS, Billingstad, Norway) was utilized, with the control results falling within the accepted reference ranges. The precision of the analytical methods was confirmed, with coefficients of variation reported at 3.3% for Se, 2.3% for Cu, and 1.8% for Zn.

The analytical quality control procedures were further supported by the Department of Bromatology at the Medical University of Bialystok, which participated in a national trace element monitoring program under the auspices of the National Institute of Public Health, the National Institute of Hygiene, and the Institute of Nuclear Chemistry and Physics in Warsaw. The serum levels of Se, Cu, and Zn obtained for the dystonia patient group were then compared with reference values established for healthy individuals.

Furthermore, the total antioxidant status (TAS) of the serum was determined through a spectrophotometric method at a wavelength of 600 nm. The assay was performed using commercial kits from Randox Laboratories Ltd. (Crumlin, UK) and measured with a UV–Vis spectrophotometer (Cintra 3030; GBC, Melbourne, VIC, Australia). The procedure involved the generation of the ABTS^®^*+ radical cation via the reaction of ABTS^®^ with peroxidase (metmyoglobin) and hydrogen peroxide. The resulting blue-green chromophore was then quantitatively analyzed using spectrophotometry, with the observed decrease in color intensity corresponding to the antioxidant concentration in the sample. The method’s accuracy was validated with a TAS Control kit, and the reference range for TAS in serum was established as 1.3–1.77 mmol/L.

## 3. Results

### 3.1. Trace Element Concentrations (Zn, Cu, Se) in Serum of Patients with Focal Dystonia

**Figure 1:**
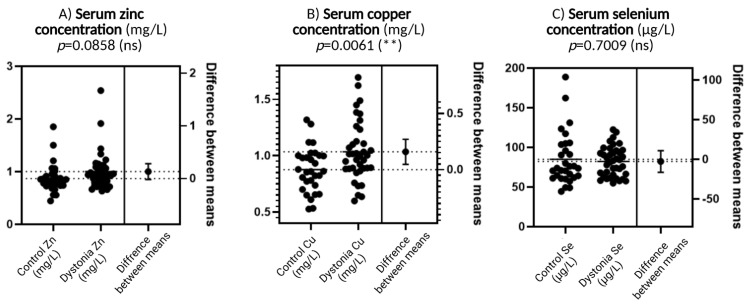
Comparison of trace element concentrations—zinc (Zn), copper (Cu), and selenium (Se)—between dystonia patients and controls. In each of the three panels, individual data are presented in the form of dot plots and the difference in means with an indication of the level of statistical significance. *N* = 30, Wilson *t*-tests were used to demonstrate differences between the control and the disease group. Statistical significance determinations: ** *p* < 0.01.

Figure 1A shows serum zinc concentration. Although there is a slightly greater spread in the results and a possible tendency for values to increase in the dystonia group compared to the control group, this difference does not reach statistical significance (*p* = 0.0858). Such a result suggests that zinc levels are not significantly different between the study groups, although there may be some direction of change that may become more apparent with more participants.

Figure 1B shows copper concentrations that differ significantly between groups. Levels are elevated in patients with dystonia compared to the control group, and this difference is statistically significant (*p* = 0.0061). This is the only one of the three elements for which a high level of significance was achieved. Elevated copper concentrations may be biologically significant because, although this element plays an important role as a cofactor of antioxidant enzymes, in excess, it can act pro-oxidatively, generating reactive oxygen species through redox reactions.

Figure 1C concerns the selenium concentration. The values in both groups are very similar, and the result of the statistical analysis (*p* = 0.7009) confirms the absence of significant differences. This suggests that selenium, despite its important function in antioxidant defense as a component of glutathione peroxidase, is not significantly altered in dystonia compared to healthy subjects.

### 3.2. Biochemical Parameters Related to Oxidative Stress (TAS, TOS, OSI) in Serum of Patients with Focal Dystonia

**Figure 2:**
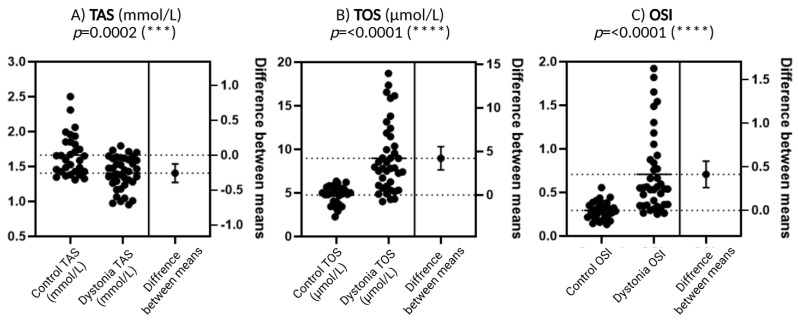
Comparison of biochemical parameters related to oxidative stress between a group of patients with dystonia and a control group. Total antioxidant capacity (TAS), total oxidative status (TOS), and oxidative stress index (OSI) were included in the analysis. *N* = 30, Wilson *t*-tests were used to demonstrate differences between the control and the disease group. Statistical significance determinations: *** *p* < 0.001; **** *p* < 0.0001.

Figure 2A presents the results for TAS, which is defined as the total antioxidant capacity of serum. Patients diagnosed with dystonia exhibited reduced values of this parameter in comparison to the control group, a discrepancy that attained a high level of statistical significance (*p* = 0.0002). This result may be indicative of an impaired antioxidant defense system in this group of patients, which would suggest a greater susceptibility to free radicals and other pro-oxidant factors.

Figure 2B presents TOS values, or total oxidative status, which reflects plasma oxidant levels. TOS levels are significantly elevated in patients with dystonia compared to the control group, with a highly statistically significant difference (*p* < 0.0001). Such a significant increase in TOS may imply that there is excessive production of reactive oxygen species (ROS) or other oxidants in the bodies of dystonia patients, which exacerbates oxidative stress.

As illustrated in Figure 2C, the OSI (Oxidative Stress Index) is calculated as the quotient of TOS (Total Oxidative Stress) and TAS (Total Antioxidant Status). This is a synthetic parameter that indicates the balance between oxidation and antioxidation processes. The OSI demonstrates a heightened level of statistical significance (*p* < 0.0001) in dystonia patients when compared to the control group. This finding suggests that the oxidation–antioxidation balance is disrupted in patients with dystonia, exhibiting a predominance of pro-oxidant factors.

Summary of all variables is provided (Table 1).

### 3.3. Gender Dependence of Parameters (Men/Women)

**Figure 3:**
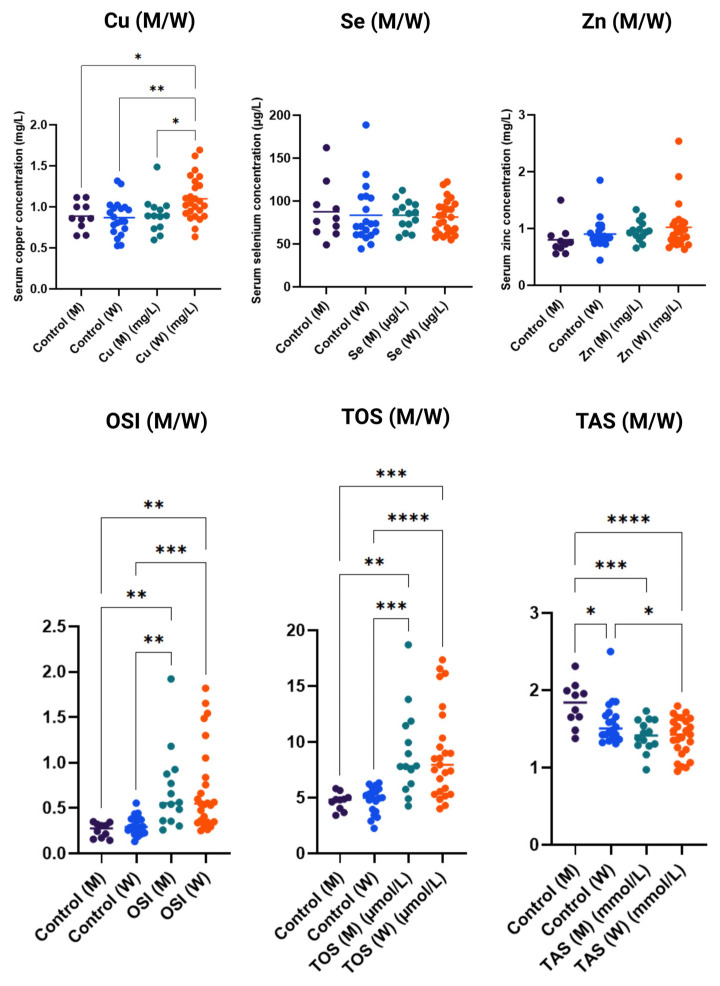
Concentrations of copper (Cu), selenium (Se), and zinc (Zn) and markers of oxidative stress (OSI—oxidative stress index, TOS—total oxidative status, TAS—total antioxidant status) in serum of men (M) and women (W) in control and study groups. Data are presented as individual measurement values along with the group average (horizontal lines). Statistical significance determinations: * *p* < 0.05; ** *p* < 0.01; *** *p* < 0.001; **** *p* < 0.0001.

Figure 3 shows serum concentrations of microelements and oxidative stress markers in the studied women (W) and men (M), compared to control groups. For copper (Cu), significantly higher concentrations were observed in women in the study group compared to men and the control group, which may indicate increased accumulation of this element in women under the analyzed conditions. Serum levels of selenium (Se) and zinc (Zn) showed considerable variability among individuals; however, no significant differences were noted between the control and study groups, regardless of sex, suggesting that the levels of these elements were not the main factors differentiating the groups.

In contrast, the analysis of oxidative stress markers revealed clear differences. OSI values were significantly higher in women in the study group compared to men and the control group, indicating increased oxidative processes in women under the analyzed conditions. Similarly, TOS was significantly elevated in the study groups compared to controls, particularly in women, suggesting enhanced oxidative stress. For TAS, a significant reduction in values was observed in the study groups, especially among women, indicating decreased antioxidant defense capacity.

### 3.4. 24-H Nutritional Interviews of Patients with Dystonia

Twenty-four-hour dietary interviews were conducted only in a group of patients with dystonia (*n* = 32) to estimate the average daily intake of selected prooxidative components (Fe, Cu, Mn) and antioxidative components (Zn, vitamin C, vitamin E, beta-carotene). The results indicated that the average intake of iron was 9.37 mg/day, copper 1.07 mg/day, and manganese 3.72 mg/day. The intake of these elements was at or above the recommended levels (RDA/AI), suggesting no significant risk of deficiency.

**Figure 4:**
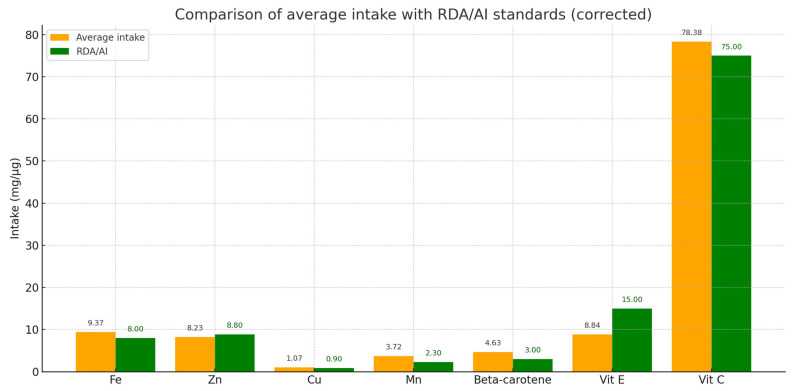
Comparison of average daily intake of selected prooxidants and antioxidants with reference values (RDA/AI) in the study group. Average intakes (orange bars) and reference values (green bars) are shown for iron (Fe), zinc (Zn), copper (Cu), manganese (Mn), vitamin E, vitamin C, and beta-carotene. The results indicate that most intakes meet or exceed the recommended values.

Regarding antioxidants, the average intake of zinc was 8.23 mg/day, vitamin E 8.84 mg/day, vitamin C 78.38 mg/day, and beta-carotene 4.63 mg/day. These values were consistent with recommended standards, indicating sufficient intake of antioxidants in the participants’ diets, which may support protection against oxidative damage from free radicals (Figure 4).

Dietary anomalies beyond prooxidants and antioxidants included protein and sodium intake. The average protein intake was 76.9 g/day, exceeding both the EAR (~54 g) and RDA (~66.7 g). Phosphorus intake was similarly high (1141 mg/day compared to an EAR of 580 mg and an RDA of 700 mg), which may raise concerns in individuals with impaired kidney function. Vitamin B6 intake was 137.5 mg/day, falling between the EAR (95 mg) and RDA (150 mg), but close to the upper limit of the recommended range. Excessive intake of phosphorus and protein over recommended levels could negatively affect kidney health and mineral balance in the long term.

The average sodium intake in the study group was 3200 mg/day, significantly exceeding the recommended AI (~1400 mg/day). Excessive sodium consumption is a well-established risk factor for developing hypertension and cardiovascular diseases, highlighting the need for nutritional education in this area.

Conversely, the intakes of vitamin D (181 IU/day with an RDA of 130 IU) and zinc (8.23 mg/day with an RDA of 8.8 mg) were in line with or close to the recommended levels, indicating adequate intake of these nutrients in the participants’ diets.

## 4. Discussion

Dystonia is a complex movement disorder characterized by involuntary muscle contractions that result in abnormal postures or repetitive movements. Although the etiology of dystonia is varied and includes both genetic and environmental factors, increasing evidence points to the important involvement of oxidative stress in the pathomechanism of this condition. In the context of neurodegeneration and functional impairment of the central nervous system, the balance between prooxidants and antioxidants plays a pivotal role in maintaining neuronal integrity and function [16]. It is evident that biochemical parameters, encompassing TOS, TAS, OSI, and copper levels, offer valuable insights into the redox status of the body. These parameters have been observed to exhibit notable variations between healthy individuals and patients afflicted with dystonia.

Serum TAS levels were found to be significantly reduced in patients, suggesting impaired endogenous antioxidant mechanisms. TAS is a measure of the total capacity of circulating antioxidants to neutralize free radicals and other reactive oxygen species (ROS). A decline in this parameter may be indicative of chronic oxidative stress, precipitated by continuous exposure of nerve cells to ROS. In the context of dystonia, oxidative damage may affect the functioning of subcortical structures such as the caudate nucleus, shell, or globus pallidus, which are involved in the regulation of muscle tone and voluntary movements [17].

Conversely, the TOS parameter exhibited a marked increase in patients with dystonia, suggesting an elevated presence of prooxidant molecules in the serum. ROS and other oxidants have been demonstrated to initiate a series of detrimental processes, including lipid peroxidation, cell membrane damage, protein denaturation, and DNA damage [18]. These processes have been shown to lead to disturbances in nerve cell metabolism and function [19]. In the absence of an adequate antioxidant response, elevated ROS have been shown to contribute to neurodegeneration or functional impairment, even in the absence of a classic degenerative picture, as may be observed in cases of dystonia.

OSI, as a ratio of TOS to TAS, is an integrated measure of oxidative stress. In the present study, OSI was found to be significantly elevated in patients with dystonia. A high OSI is indicative of a substantial shift in the redox balance, favoring prooxidant factors. Numerous reports in the scientific literature have documented the role of oxidative stress in neurological diseases, including Parkinson’s disease [20,21], multiple sclerosis, and Alzheimer’s disease [22,23]. In the case of dystonia, there have been fewer studies to date [13,24]; however, the results of the present study confirm that disturbances within the redox economy may also have pathogenetic significance in this nosological entity.

These findings suggest the potential for oxidative stress markers to serve as diagnostic or prognostic biomarkers in dystonia. Moreover, the observed changes indicate that antioxidant therapy could be a potential adjunct to the treatment of dystonia, particularly in patients in whom mechanisms related to mitochondrial dysfunction and oxidative stress predominate.

However, several limitations of the present study should be acknowledged. First, the sample size may not have been sufficient to detect subtle associations or to generalize findings to the broader population of dystonia patients. Second, potential confounding factors such as comorbid conditions and lifestyle differences (e.g., physical activity, smoking) may have influenced redox status but were not fully controlled. Additionally, although dietary intake was reported to be generally balanced, it was not quantitatively assessed, which limits the interpretation of nutritional impact on oxidative stress levels.

Although the diet of patients with dystonia in the study group was generally characterized by an appropriate intake of key oxidative components based on available information, the lack of precise dietary quantification represents a potential confounding factor that warrants further investigation.

## 5. Conclusions

The results provide important indications of an oxidant–antioxidant imbalance in focal dystonia. The observed decline in TAS levels, coupled with the concurrent rise in TOS and OSI values, provides evidence of an escalating oxidative stress burden throughout the disease. Elevated copper levels, an element that can act pro-oxidatively in excess, may be important in the mechanisms of neuronal damage. Despite the absence of a statistically significant divergence in zinc and selenium levels between the groups, the observed increase in zinc concentrations within the patient group may indicate ongoing alterations in its homeostasis.

## Figures and Tables

**Table 1:** Summary of mean ± SD and median values with extreme values in dystonia patients.

	Mean	SD	Minimum	Maximum
Zn	1.000	0.3506	0.6320	2.538
Cu	1.034	0.2633	0.5980	1.694
Se	82.24	18.56	54.85	122.4
TAS	1.404	0.2320	0.9540	1.795
TOS	8.982	3.956	4.000	18.70
OSI	0.7075	0.4583	0.2500	1.922

## Data Availability

Data are unavailable due to privacy restrictions.

## Abbreviations

The following abbreviations are used in this manuscript:

Cu	Copper
Zn	Zinc
OSI	Oxidative Stress Index
TOS	Total Oxidant Status
TAS	Total Antioxidant Status
ROS	Reactive Oxidative Species
RDA	Recommended Dietary Allowance
AI	Adequate Intake
